# Interleukin-6 receptor in the recent-onset rheumatoid arthritis

**DOI:** 10.1186/1479-5876-9-S2-P39

**Published:** 2011-11-23

**Authors:** Marco Antonio Montes-Cano, Sonsoles Reneses, Jose Raul García-Lozano, Fuensanta Torrecillas, Cristina Abad-Molina, Alicia García, Antonio Núñez Roldán, Maria Francisca González-Escribano

**Affiliations:** 1 Servicio de Inmunología, Hospital Virgen del Rocío/IBIS, Sevilla, Spain

## Introduction

The α subunit of the soluble receptor interleukin-6 (sIL-6R) is mainly generated by shedding of the membrane-bound form. This process is influenced by a functional polymorphism (rs 2228145 A>C) resulting in a substitution of aspartic acid to alanine (D358A) at the proteolytic cleavage site.

## Objective

To investigate the influence of the rs2228145 polymorphism in sIL-6R plasma levels.

## Patients and methods

A total of 141 RA patients and 206 unrelated healthy individuals matched by age and gender were included in the study. RA patients were diagnosed according ACR criteria and they had less than 1 year of evolution at the study time. The sIL-6R protein levels were determined by an ELISA quantitative sandwich and the rs2228145 genotyping was performed using a Taqman assay. Non parametric tests (U Mann-Whitney and Kruskal-Wallis) were used for median comparison and the chi-square test for comparison of the genotypic distribution.

## Results

The sIL-6R median concentration was lower in patients than in healthy control group (164.4 ng/mL vs. 185.3 ng/mL, p=0.0002). Moreover, median of sIL-6R concentration was different according with the rs2228145 genotype in both groups, RA patients (AA: 135.8, AC: 189.3 and CC: 233.0, p <0.0001) and controls (AA: 142, AC: 200.2 and CC: 242.7, p <0.0001). When the patients and controls were stratified according to their genotypes (AA and AC+CC), the sIL-6R median concentration was lower in both patient groups compared with their corresponding healthy controls (p=0.01 for AA and p=0.04 for AC+CC). Finally, no differences in the distribution of the rs2228145 genotypes were found on comparing patient and control groups (p=0.18).

## Conclusions

The functional Asp358Ala polymorphism influences the sIL-6R concentration. Nevertheless, additional factors are necessary to explain the lower sIL-6R concentration found in patients since the differences among both groups remain after stratification by genotypes.

